# Radiotherapeutic treatment options for oligotopic malignant liver lesions

**DOI:** 10.1186/s13014-021-01779-5

**Published:** 2021-03-16

**Authors:** Peter Wust, Marcus Beck, Robert Dabrowski, Oliver Neumann, Sebastian Zschaeck, David Kaul, Dominik P. Modest, Carmen Stromberger, Bernhard Gebauer, Pirus Ghadjar

**Affiliations:** 1 Charité – Universitätsmedizin Berlin, corporate member of Freie Universität Berlin and Humboldt-Universität zu Berlin, Department of Radiation Oncology, Augustenburger Platz 1, 13353 Berlin, Germany; 2 Berlin Institute of Health (BIH), Anna-Louisa-Karsch-Straße 2, 10178 Berlin, Germany; 3 Charité – Universitätsmedizin Berlin, corporate member of Freie Universität Berlin and Humboldt-Universität zu Berlin, Department of Medical Oncology, Berlin, Germany; 4 Charité – Universitätsmedizin Berlin, corporate member of Freie Universität Berlin and Humboldt-Universität zu Berlin, Department of Radiology, Berlin, Germany

**Keywords:** Liver metastasis, Oligometastasis, Brachytherapy, Radiation therapy, Colorectal cancer

## Abstract

**Background:**

Several radiotherapeutic approaches for patients with oligotopic malignant liver lesions unfit for surgical resection exist. The most advanced competitive techniques are high-dose-rate (HDR) brachytherapy, Cyberknife, volume-modulated-arc therapy (VMAT) and Tomotherapy. We evaluated the optimal technique by a planning study for a single ablative dose with different lesion sizes.

**Methods:**

We compared dose distributions of HDR-brachytherapy with stereotactic ablative radiotherapy using the Cyberknife, VMAT or Tomotherapy. Tumor-control-probabilities (TCP), normal-tissue-complication-probabilities (NTCP) were determined in a theoretical framework applying a single dose of 20 Gy (demanding 95% coverage) for intrahepatic lesions of 1–5 cm in size. We evaluated therapeutic ratios by TCP (mean dose in the lesion) relative to high-dose (conformality) or low-dose liver exposition in dependency on the lesion size for each technique. In addition, we considered treatment times and accuracy (clinical target volume vs planning target volume).

**Results:**

HDR-brachtherapy has the highest therapeutic ratios with respect to high-dose as well as low-dose liver exposition even for extended lesions, and the Cyberknife being suited second best. However, for lesions ≥ 3 cm diameter the therapeutic ratios of all ablative techniques are increasingly converging, and better tolerance and shorter treatment times of noninvasive external techniques become more important. On the other hand, mean tumor doses of HDR-brachytherapy of near 60 Gy are unattainable by the other techniques gaining only 22–34 Gy, and the conformality of HDR-brachytherapy is still rather good for lesions ≥ 3 cm diameter.

**Conclusions:**

HDR-brachytherapy is by far the most effective technique to treat intrahepatic lesions by a single fraction, but sparing of the surroundings declines with increasing lesion size and approaches the benchmarks of external beam radiosurgery techniques. External beam radiotherapy has the advantage to use suitable fractionation schedules.

## Background

The liver is a common target for metastases from various solid cancers, especially from colorectal cancer and other gastrointestinal cancers. Surgical resection of metastases has been shown to improve survival in colorectal cancer [1], thus surgery is regarded as the preferred local treatment strategy for oligotopic colorectal cancer liver metastases. However, only less than one third of patients are appropriate candidates for surgical resection due to location and distribution of lesions within the liver, patient co-morbidities prohibiting surgical intervention or extrahepatic involvement [2, 3]. Despite major advances in systemic treatments for colorectal cancer during the last decades still long-term control of liver disease is almost impossible without additional local treatment of metastatic disease [4]. Therefore, other local treatment strategies for patients with oligotopic malignant liver lesions are warranted to complement systemic therapies in cases not qualifying for surgical resection [3, 5]. There are some general restrictions to the use of radiofrequency ablation (RFA) and other thermoablative procedures, which include danger to thermosensitive structures, limitations to treat lesions in proximity of larger vessels due to cooling, ineffectivity to treat lesions with increased arterial tumor perfusion such as hepatocellular carcinoma or neuroendocrine tumors and limited local control of lesions > 3 cm size [5, 6].

Image-guided high-dose-rate interstitial (HDR-) brachytherapy uses an iridium-192 source of < 1 mm diameter, which is moved in an implanted catheter array according to a treatment plan in order to generate an optimal dose distribution for a specified target volume and adjacent organs of risk. It overcomes the mentioned limitations of RFA [6] and was shown to be an effective treatment option [7–10]. Likewise, a variety of modern external beam radiation techniques are available using dedicated linear accelerators, competing with the interstitial rather accurate but invasive method. This includes intensity modulation radiotherapy (IMRT), rotational techniques such as volume modulated arc therapy (VMAT) or Tomotherapy and the Cyberknife, which are suitable for radiosurgery in a single fraction [3, 11].

Other non-surgical treatment options do exist for advanced unresectable liver metastases including transarterial chemoembolization, selective internal radiation therapy and hepatic arterial infusion, which are mainly useful for disseminated liver disease. In this article, we focus on oligotopic malignant liver lesions that are treated in a single fraction as this is a logistical and practical advantage for the patient, however without evidence of improved oncological outcome compared to fractionated schedules.

It is the task of the radiation oncologist to ascertain a well-suited radiotherapy technique for a given target volume embedded in an individual anatomy with specified organs of risk or regions of interest (ROI). This article compares radiotherapeutic single dose approaches such as HDR-brachytherapy, rotational techniques (VMAT, Tomotherapy) and Cyberknife. For that reason we calculated and analyzed optimal dose distributions for a single dose of 20 Gy covering intrahepatic lesions between 1 and 5 cm diameter outlining the pros and cons for these different techniques.

## Methods

We specified intrahepatic spherical liver lesions in a peripheral location of 1–5 cm diameter and segmented adjacent ROI (liver, right/left kidney, stomach, heart and right lung) to estimate toxicity. For all approaches treatment planning dose calculations were optimized demanding mean doses D_mean_ as high as possible inside the lesions ensuring the prescribed standard dose of 20 Gy with target coverage of > 95% and minimal burden to the liver. Listed parameters include D_mean_ in the lesions characterizing the TCP, and V_10Gy_, V_5Gy_, V_2Gy_ and V_1Gy_ of the liver, and maximum doses D_max_ of other critical organs. The parameters for any other prescribed dose can easily be calculated by rule of three.

The following four radiation techniques were evaluated:

- *HDR-brachytherapy* We assumed one or five accurately implanted catheters in the lesion and used the planning system Brachyvision®. The planning target volume (PTV) and clinical target volume (CTV) was set equal, because catheters do not move relative to the lesion. We note that catheter implantation requires an invasive image-guided procedure that must be accurate to the millimeter, and is demanding for patients and staff and involves some risks (bleeding, infection etc.).
- *Cyberknife* This radiosurgery system uses a compact linear accelerator on a robotic manipulator that can radiate from a large spherical angle using standard circular collimators of selected diameter. The principal advantage is the full three-dimensional performance and the integrated image guidance system that enables on-line tracking during radiation. An almost perfect tracking is implemented for Cyberknife based on a gold marker implantation that an image-guided invasive procedure similar to the catheter implantation for brachytherapy, but less critical in terms of accuracy. The PTV and CTV was set equal. An inverse planning algorithm is available.
- *Volume Modulated Arc Therapy (VMAT)* Using a standard linear accelerator (Siemens/Varian Medical Systems Inc., Palo Alto, 94304 CA, USA) with one or more arcs rotating around the iso-center typically located in the lesion with variable speed, intensity and multileaf collimator (MLC) setup. Leafs have thickness of 0.5 cm in the center (20 × 20 cm). To account for breathing-induced movement of the liver the diameter was enlarged by one cm to form the PTV. To restrict this safety margin to only 5 mm we assumed patient compliance to control respiratory motion either with shallow breathing or breath-hold techniques in addition to cone-beam CT for image guidance. Optimization was performed with respect to dose specifications for the PTV and the ROI (limiting V_X%_, volume with doses ≥ X%) by use of the planning system Eclipse®.
- *Tomotherapy* Using a binary (on/off) collimator of 1 cm thickness that allows full intensity modulation slice by slice in a dedicated linear accelerator (Accuray Inc., Sunnyvale, CA 94089, USA). Tomotherapy is superior for complex target volumes and complicated constraints, but is theoretically nearly equivalent to VMAT for simple targets like spheres. Again the CTV was enlarged by one cm in diameter to form the PTV assuming adequate control of respiratory motion and megavoltage CT for image guidance. The planning system Tomoplan® calculates optimized dose distributions with respect to the selected restrictions.

We used the Lyman/Kutcher/Brown (LKB) model to estimate normal tissue complication probabilities (NTCP) for the liver [12–16] in order to evaluate long-term toxicity. In short, the LKB-model exploits the tolerance dose TD_50/5_ implying a specified complication in 50% in 5 years after irradiation of the entire organ. This dose is the mean of a Gaussian distribution of tolerance doses with standard deviation σ = m × TD_50/5_. Integration over this probability function yields the cumulative distribution function that is the sigmoid NTCP-curve. The parameter m depends on the critical organ (here liver) and determines the slope of the NTCP-curve. Another crucial parameter is n describing the volume dependencies of the tolerance doses. The partial volume is υ_i_ = V_i_/V_0_ with V_0_ the volume of the entire organ. Then the tolerance dose TD_50/5_(υ_i_) of the partial volume is larger than TD_50/5_(υ_0_ = 1) and can be calculated utilizing the volume parameter n by the formula TD_50/5_(υ_i_) = TD_50/5_(1)/υ_i_^n^.

For better comparison we calculated therapeutic ratios using the term D_mean_ (lesion d)/(%liver > 10 Gy) comparing TCP with high-dose exposition (conformality) and the term D_mean_ (lesion d)/(%liver > 1 Gy) comparing TCP with low-dose exposition (long-term risk).

## Results

**Fig. 1:**
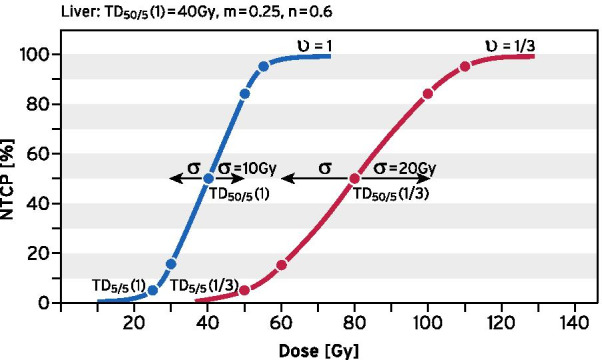
NTCP-curves for the liver according to LKB-model and parameters of Table 1. A large volume dependency is found for n = 0.6, if we compare irradiation of the entire organ (1) with irradiation of only one third (1/3)

Table 1 is listing the volume depending parameters TD_5/5_, TD_50/5_, σ (for the entire volume 1 or a partial volume 1/3) as well as m, n and α/β for various organs or tissues in comparison to the liver. The tolerance doses TD and their volume dependencies assume a conventional fractionation [17]. By using the LKB-model and Table 1 we constructed the NTCP-curves for the liver in Fig. 1 with important reference points for illustration [18].

**Table 1:** Parameters to calculate normal tissue complication probabilities (NTCP) for conventionally fractionated regimens

Organ Tissue	TD_5/5_(1) [Gy]	TD_5/5_(1/3) [Gy]	TD_50/5_(1) [Gy]	TD_50/5_(1/3) [Gy]	σ [Gy] (1)	σ [Gy] (1/3)	n (volume)	m (slope)	α/β [Gy]	Clinical endpoint
Liver	25	50	40	80	10	20	0.6	0.25	1.5	Radiation-induced liver disease (RILD)
Lung	18	45	25	74	12	15	0.9	0.2	2.5	Pulmonary dysfunction
Kidney	23	50	28	65	3	6.5	0.7	0.1	0.5–4	Nephropathy/renal failure
Small bowel	40	50	55	70	10	14	0.1–0.2	0.2	3–5	Obstruction/perforation
Stomach	45	54	60	76	10	11	0.1–0.2	0.15	5	Bleeding/ulcer
Brain	45	60	60	86	12	17	0.2–0.3	0.2	2	Necrosis/cognitive dysfunction
Heart	15	18	29	35	12	14	0.16	0.4	3	Cardiac mortality
Skin	55	65	65	79	6.5	8	0.06–0.16	0.1	1–4	Fibrosis/ulcer/Teleangiectasia
Spinal cord	47	50	70	74	14	15	0.05	0.2	2.5–5	Myelopathy
Nerves Plexus	60	62	100	104	30	31	0.03	0.3	1.5–3.5	Neuropathy/plexopathy

TD_5/5_: mean dose leading to a complication in 5% within 5 years after irradiation of the entire organ (1) or one third (1/3); _TD50/5_: mean dose leading to a complication in 50% within 5 years after irradiation of the entire organ (1) or one third (1/3); σ = m × TD_50/5_

We note that m = 0.25 for the liver is in the upper range, i.e. the tolerance doses are not as sharply defined. In consequence, for a rather moderate dose of 10 × 2 Gy → 20 Gy (TD_50/5 _− 2σ) to the whole liver we expect a radio-induced liver disease (RILD) in more than 2%. On the other hand, n = 0.6 is relatively high, i.e. for partial volumes we achieve much higher tolerance doses. For a partial liver irradiation of one third of the liver the TD_5/5_(1/3) is shifting to 50 Gy and the TD_50/5_(1/3) even to 80 Gy.

We used the linear quadratic model to calculate biologically effective doses BEQ_2Gy_ (assuming conventional fractionation with daily doses d = 2 Gy) iso-effective to single doses D_S_ for tissues with different α/β values [19] in order to compare the abovementioned techniques that apply high single doses in circumscribed lesions (Table 2):

$${\text{BEQ}}_{{2{\text{Gy}}}} = \, (\upalpha /\upbeta + {\text{D}}_{{\text{S}}} /(\upalpha /\upbeta + \, 2) \times {\text{D}}_{{\text{S}}}$$

**Table 2:** Biologically effective doses applied by conventionally fractionated regimens BEQ_2Gy_ (5 × 2 Gy with d = 2 Gy) in dependency on α/β that are isoeffective to a single dose D_S_

D_S_ [Gy]	BEQ_2Gy_ [Gy] α/β = 1.5 Gy	BEQ_2Gy_ [Gy] α/β = 2 Gy	BEQ_2Gy_ [Gy] α/β = 3 Gy	BEQ_2Gy_ [Gy] α/β = 5 Gy	BEQ_2Gy_/BED [Gy] α/β = 10 Gy
5	10	9	7	5	3/8
8	23	20	16	11	7/14
10	34	30	24	17	10/20
12	48	42	34	24	14/26
15	73	64	51	36	21/38
18	103	90	72	51	30/50
20	126	110	88	63	37/60
25	189	169	140	107	73/88
30	274	225	192	137	80/120
35	365	324	266	200	131/158

For comparison the BED (for d → 0) are shown for α/β = 10 Gy

D_s_, single dose; BEQ_2Gy_, isoeffective cumulative dose for conventionally fractionated regimens of 5 × 2 Gy per week; BED, biologically effective dose for a low-dose rate regimen with d → 0

In the last column for α/β = 10 Gy we added the iso-effective biologically effective doses BED (assuming a low-dose-rate regimen with d → 0), for easier comparison with published data (see “Discussion” section): BED = [(α/β + D_S_/α/β] × D_S_.

The liver is particularly sensitive to large single doses because of the low α/β = 1.5 Gy. Therefore, only two fractions of 5 Gy to the entire liver (iso-effective to 20 Gy conventionally fractionated) might induce a RILD in as much as 2% of patients.

**Fig. 2:**
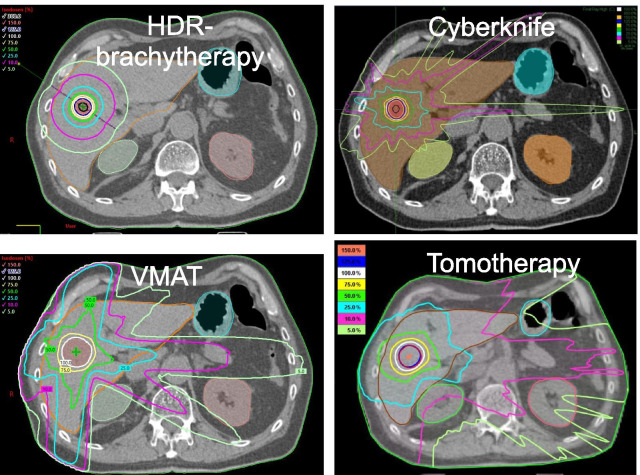
Dose distributions comparing the four radiotherapy techniques, if the standard single dose 20 Gy is given to a 2 cm lesion. The isodoses range from 150% (30 Gy, orange), 100% (20 Gy, white), 75% (15 Gy, yellow), 50% (green, 10 Gy), 25% (5 Gy, light blue), 10% (2 Gy, purple) to 5% (1 Gy, light green) according to the inserted scales. The ROIs are also shown such as liver (brown), stomach (blue) and kidneys (green, red). For such a small lesion HDR-brachytherapy is clearly superior in comparison to the external techniques

The dose distributions of the considered treatment techniques applied to a lesion with d = 2 cm are depicted in Fig. 2. Important DVH parameters for the lesions and the ROI liver, right kidney and stomach are listed in Table 3 for lesion sizes d = 1, 2, 3, 5 cm.

**Table 3:** Results of the planning study for comparison (see text)

Technique	HDR-Brachytherapy (CTV ≡ PTV)	Cyberknife (CTV ≡ PTV)	VMAT (d_HDR_ + 1 cm)	Tomotherapy (d_HDR_ + 1 cm)
Lesion ∅ 1 cm/2 cm (0.7 ml/5.2 ml)	1 catheter			
Irradiation time [min]	0.9/3.2	72/51	2.6/2.3	3.0/4.2
D_mean_ [Gy]	56.3/58.1	28.6/34.2	23.9/22.8	25.5/28.2
Coverage 20 Gy [%]	100/98	99.6/99	100/98	100/100
Liver mean dose [Gy]	0.3/1.1	0.7/1.2	1.0/1.7	1.4/2.5
Volume > 10 Gy [ml (%)]	0.5 (0.04)/6 (0.45)	1.3 (0.1)/9.4 (0.7)	17.5 (1.3)/54 (4)	25 (1.9)/77 (5.8)
Volume > 5 Gy [ml (%)]	2 (0.2)/18 (1.3)	8.4 (0.6)/62 (4.6)	94 (7.0)/165 (12)	100 (7.5)/236 (17.7)
Volume > 2 Gy [ml (%)]	24 (1.8)/161 (12)	58 (4)/248 (19)	187 (14)/282 (21)	360 (27)/466 (35)
Volume > 1 Gy [ml (%)]	71 (5)/417 (31)	220 (16)/444 (33)	269 (20)/376 (28)	413 (31)/560 (42)
Kidney right D_max_ [Gy]	0.4/1.2	1.1/3.4	2.6/3.7	3.0/4.6
Stomach D_max_ [Gy]	0.1/0.2	0.8/1.4	0.5/1.4	1.3/2.1

∅ 3 cm lesion (16.5 ml)	1/5 catheters			
Irradiation time [min]	6.9/6.4	64	2.1	5.1
D_mean_ [Gy]	58.2/50.1	31.7	21.5	32.5
Coverage 20 Gy [%]	98/96	97.5	93	99
Liver mean dose [Gy]	2.1/2.0	2.0	2.3	4.4
Volume > 10 Gy [ml (%)]	24 (1.8)/22 (1.6)	24 (1.8)	81 (6.0)	187 (14)
Volume > 5 Gy [ml (%)]	59 (4.4)/54 (4.0)	138 (10.5)	207 (15.4)	373 (28)
Volume > 2 Gy [ml (%)]	443 (33)/416 (31)	460 (35)	403 (30)	600 (45)
Volume > 1 Gy [ml (%)]	887 (66)/860 (64)	657 (50)	598 (40)	733 (55)
Kidney right D_max_ [Gy]	2.7/2.5	3.9	8.5	6.4
Stomach D_max_ [Gy]	0.5/0.5	2.2	0.9	3.1

∅ 5 cm lesion (75.9 ml)	1/5 catheters			
Irradiation time [min]	18.8/17.4	53	1.8	6.1
D_mean_ [Gy]	58.7/46.3	34.2	22.1	31.3
Coverage 20 Gy [%]	98/98	99.4	97	99
Liver mean dose [Gy]	4.7/4.6	4.4	4.1	5.2
Volume > 10 Gy [ml (%)]	128 (9.5)/120 (9.0)	126 (10)	159 (12)	253 (19)
Volume > 5 Gy [ml (%)]	443 (33)/416 (31)	446 (36)	417 (31)	653 (49)
Volume > 2 Gy [ml (%)]	1102 (82)/1048 (78)	791 (63)	712 (53)	773 (58)
Volume > 1 Gy [ml (%)]	1304 (97)/1290 (96)	937 (75)	874 (65)	959 (72)
Kidney right D_max_ [Gy]	2.7/2.5	3.9	9.2	8.9
Stomach D_max_ [Gy]	0.5/0.5	2.8	2.9	2.7

*CTV* clinical target volume, *PTV* planning target volume, *D*_*max*_ maximal dose, *D*_*mean*_ mean dose, *HDR* high-dose-rate, *VMAT* volumetric modulated arc technique

HDR-brachytherapy is by far most effective gaining intralesional mean doses D_mean_ > 55 Gy, whereby increasing the number of catheters (5 vs 1 catheter) homogenizes the intralesional dose distribution and lowers D_mean_. We achieved much lower D_mean_ of approximately 30 Gy by Cyberknife and Tomotherapy and only 22 Gy by VMAT. For lesion sizes < 3 cm the rather high TCP is combined with improved conformality in the high-dose range around 10 Gy, sparing of the liver in the low-dose range around 1–2 Gy and low exposition of the other surrounding organs (e.g. right kidney, stomach) in comparison to all external radiation techniques. For lesions > 3 cm D_mean_ is still superior for HDR-brachytherapy, but dose sparing in liver and surroundings is continuously declining with increasing size. For lesions of 5 cm size the low dose exposition with 1–2 Gy is even higher for HDR-brachytherapy in comparison to all external techniques and equal for exposition with 5 Gy, but the strikingly high dose to the tumor remains. Interestingly, more catheters (5 vs 1 catheter) neither improve the effectiveness nor the sparing of surroundings.

HDR-brachytherapy achieves the highest conformality for small lesions that also the dose distribution reflects (Table 3, Fig. 2). For example for the 1 cm lesion only 0.5 ml healthy liver is exposed to > 10 Gy versus 1.3 ml for Cyberknife and around 20 ml for VMAT/Tomotherapy. Also the sparing of the liver in the low dose range is excellent, e.g. for the 1 cm lesion only 70 ml healthy liver exposed to 1 Gy versus 200–400 ml with external techniques. We emphasize that these advantages require a nearly perfect positioning of the catheter(s). For all lesion sizes the conformality of the Cyberknife is between HDR-brachytherapy and VMAT/Tomotherapy, but low dose expositions (volume > 1–2 Gy) are in the same range for all external techniques and higher than for interstitial techniques.

The VMAT technique that is standard in modern linear accelerators performs with lower intralesional mean doses of ~ 22 Gy accompanied by the lowest liver exposition at 1 Gy for lesions ≥ 3 cm. Tomotherapy achieves higher intralesional mean doses ~ 30 Gy with a larger load to the surroundings accordingly. The isodoses for higher doses (5–10 Gy) are more compact for Tomotherapy than VMAT (Fig. 2).

**Fig. 3:**
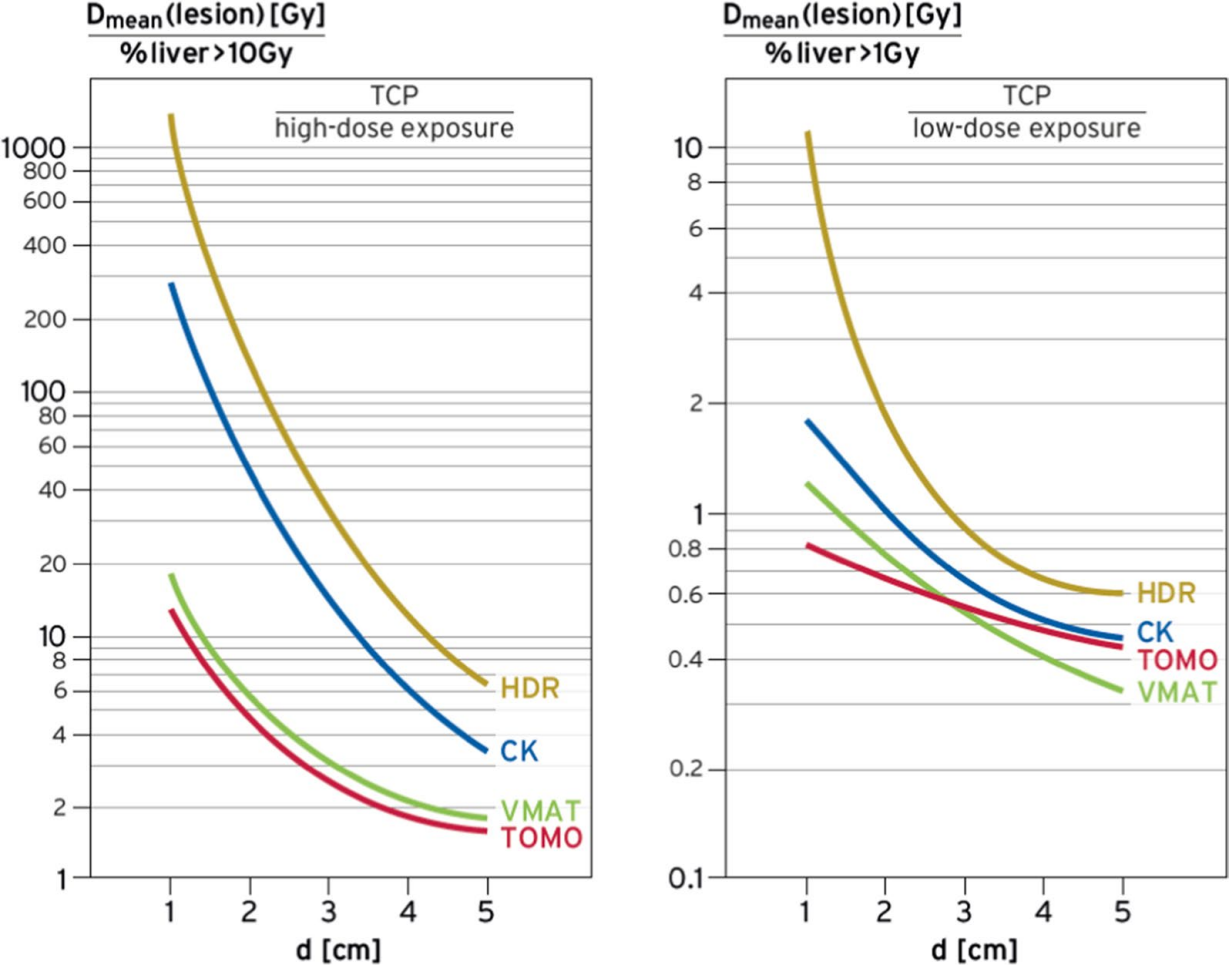
Therapeutic ratios for all the four radiation techniques relating TCP (mean dose in the lesion) either to high-dose exposure (conformality, left) or low-dose exposure (long-term risks, right) of the liver

For a conclusive comparison we plotted the therapeutic ratios of the radiotherapy techniques in dependency on lesion sizes for high intrahepatic doses > 10 Gy (Fig. 3, left) and low intrahepatic doses > 1 Gy (Fig. 3, right). The HDR-brachytherapy performs always superior, but these advantages decline with increasing lesion size. The Cyberknife comes second and the external standard techniques (VMAT, Tomotherapy) come third. While for a lesion of d = 1 cm the therapeutic ratios are far apart by two decades (high dose) or one decade (low dose), the ratios differ only by a factors 2–3 for d = 5 cm.

The performance of the (typically invasive) Cyberknife lies in between HDR-brachytherapy and VMAT/Tomotherapy, but again the advantage declines with increasing lesion size.

VMAT shows a slight advantage over Tomotherapy for small lesions, mainly due to finer leafs in the center (0.5 cm vs 1 cm), while Tomotherapy performes better for large and complex target volumes (as illustrated in Fig. 3, right).

Table 3 shows also relevant differences in the irradiation times that might influence the treatment decision. Each Cyberknife treatment session consumes about 1 h in comparison to only 2 min for VMAT and 3–6 min for Tomotherapy. In case of HDR-brachytherapy treatment time is rather short for small lesions and increases considerably with lesion size, e.g. around 20 min for lesions of 5 cm assuming a recent exchange of the iridium-source and up to 40 min, if the source change is long ago (typically about two months).

In addition, we must consider for a treatment decision that feasibility, invasiveness and risks of the techniques greatly differ.

## Discussion

We have compared four radiosurgery techniques for a standard setup with equal optimization conditions (see “Methods” section) and ascertained image-guided HDR-brachytherapy as by far the most effective radiotherapy technique to control locally oligotopic malignant liver lesions in a single session. This is an option for patients with metastases either unresectable and/or unfit for surgical resection and remains valid even for larger lesions in our planning study. However, sparing of the surroundings by HDR-brachytherapy declines with increasing lesion size and finally approaches the benchmarks of external beam radiation techniques. Notably, HDR-brachytherapy generates an inhomogeneous dose distribution with dose excesses in the target around the catheters achieving intralesional mean doses of > 55 Gy. This is important as rather high single doses are estimated to be required for reliable and long-term tumor control.

Ricke et al. [9] conducted a dose escalation study comparing prescribed target doses of 15 Gy, 20 Gy versus 25 Gy. They found a long-term local control (> 3 years) in 95% for 25 Gy (BED = 88 Gy) and only 65% for 20 Gy (BED = 60 Gy) according Table 2. Local control was also correlated with D_min_ (> 20.5 Gy) indicating that the coverage is important because of the steep dose gradient at the tumor margin [9, 20]. Herfarth et al. 2001 [21] achieved for liver tumors/metastases lower local control of 80% for 22–26 Gy single fraction in the reference point (80% of this dose enclosing the PTV).

Numerous clinical trials with various fractionation schemes were recently reviewed (3). Generally, a BED > 100 Gy was recommended to achieve a satisfactory long-term local control. In a careful evaluation a BED near 140 Gy was estimated for a long-term (> 1 year) local control of 90% for colorectal cancer that turned out as the most resistant cancer type [22]. Such high BEDs require single doses > 30 Gy (Table 2) that is in our analysis most likely accomplished by HDR-brachytherapy.

The LQ-model confirms that rather high single doses might be necessary for local control. Table 4 illustrates that single doses of 20–30 Gy are only sufficient for radiation sensitive tumors (with α = 0.3 Gy^−1^) and/or tumors with low α/β < 10 Gy. However, radioresistant tumors (with α < 0.2 Gy^−1^ and α/β > 5 Gy) might get missed with single doses below 30 Gy. Clinical data suggest that α/β of metastases are typically > 10 Gy [21] resulting in higher single doses > 30 Gy for reliable local control. Single equivalent dose of ≥ 45 Gy achieved a two-year local control of 100% compared with 54% for < 45 Gy [11]. Our planning study shows (Table 3) that external methods have difficulty depositing such high single doses is the lesions.

**Table 4:** Tumor control with single doses D_s_ = {20.7/β + (α/2β)^2^}^1/2 ^− (α/2β) depending on α and α/β

	Radiation sensitive	Intermediate	Radiation resistant
α/β [Gy]	α = 0.3 Gy^−1^	α = 0.2 Gy^−1^	α = 0.1 Gy^−1^
20	28	37	55
10	22	28	41
5	16	20	30
2	11	13	19

For single doses around 50 Gy tumor control is typically achieved

HDR-brachytherapy performs with a steep dose gradient sparing the surrounding tissues and reducing their NTCP. Both features favor in particular HDR-brachytherapy for intrahepatic metastases or tumors, because the liver is sensitive to radiation exposure in a large volume with low or medium radiation doses. For the liver much care is required, if large volumes or even the entire organ are irradiated given the low TD_5/5_(entire liver) of 25 Gy (conventional fractionation).

CT-guided implantations of brachytherapy catheters and implantation of a gold marker for Cyberknife tracking are similar minimally-invasive procedures, sometimes comparable in the risk. In case of the Cyberknife the risk of gold marker implantation must be balanced against the benefit of enhanced precision by reducing the safety margin (taking CTV as PTV). On the other hand, the quality of HDR-brachytherapy critically depends on the precise placing of the catheters, while the geometry of gold marker(s) near or in the target is not as critical. It must be considered that HDR-brachytherapy and Cyberknife are more invasive and includes some risks, if compared with non-invasive external radiation methods (VMAT, Tomotherapy).

If we address single session treatment approaches using either HDR-brachytherapy or ablative radiosurgery, the non-invasive external radiation methods accomplish lower therapeutic ratios (Fig. 3), as they are not able to deposit sufficiently high doses in the lesion (> 30 Gy) and to spare simultaneously the surroundings. Advantages of HDR-brachytherapy and Cyberknife decline for lesions > 3 cm. It is to note as limitation that besides logistical and practical advantages of a single session treatment there is no data available showing improved oncological outcome compared to fractionated schedules. Using sophisticated treatment delivery methods, for instance a breath-hold technique, may allow the application of PTV margins < 10 mm which would improve the comparative dosimetry of the non-invasive external radiation methods. Further, we did not compare outcome data of HDR-brachytherapy with RFA and/or other thermoablative procedures.

Various fractionation schedules have been applied using external radiation techniques including the Cyberknife. For intralesional BED > 100 Gy the use of fractionated stereotactic ablative radiotherapy SABR is common (3). An optimal SABR-schedule of 3 × 16 Gy was suggested [11] yielding BED = 125 Gy (for α/β = 10 Gy). Another analysis recommended 3 × 17 Gy for colorectal cancer metastases with BED = 138 Gy [22].

The possibility of SABR using fractionated schedules is an important asset for the non-invasive radiation techniques [23, 24], which is particularly useful for larger lesions. On the other hand, treatment of a few circumscribed lesions in a single session appears attractive as well. The choice between the various options might finally depend on patient preferences.

## Conclusions

HDR-brachytherapy is attractive to treat oligotopic liver metastases in a single session, because it is maximally effective and spares nevertheless the liver yielding the highest therapeutic ratios. Repeated treatments are therefore possible in case of recurrent metastases. VMAT and Tomotherapy are of interest for lesions ≥ 3 cm, because liver exposure becomes comparable to HDR-brachytherapy but tolerance and patient comfort are much better. In case of a single dose advantages of Cyberknife are limited, because it requires invasive procedures and long treatment times, but is inferior to HDR-brachytherapy. VMAT and Tomotherapy are comparable with slight advantages of VMAT for small lesions and improving performance of Tomotherapy for larger lesions. However, all external beam radiotherapy approaches, including Cyberknife, have the important advantage to enable any kind of fractionated schemes.

## Data Availability

The datasets used and/or analyzed during the current study are available from the corresponding author on reasonable request.

## Abbreviations

HDR: High-dose-rate
VMAT: Volume-modulated-arc therapy
NTCP: Normal-tissue-complication-probabilities
RFA: Radiofrequency ablation
IMRT: Intensity modulation radiotherapy
SABR: Stereotactic ablative radiotherapy
PTV: Planning target volume
CTV: Clinical target volume
ROI: Region of interest
DVH: Dose volume histograms

## References

[1] Morris EJ, Forman D, Thomas JD (2010). Surgical management and outcomes of colorectal cancer liver metastases. Br J Surg.

[2] Hewish M, Cunningham D (2011). First-line treatment of advanced colorectal cancer. Lancet.

[3] Romesser PB, Neal BP, Crane CH (2021). External beam radiation therapy for liver metastases. Surg Oncol Clin N Am.

[4] Jawed I, Wilkerson J, Prasad V (2015). Colorectal cancer survival gains and novel treatment regimens: a systematic review and analysis. JAMA Oncol.

[5] Wong SL, Mangu PB, Choti MA (2010). American Society of Clinical Oncology 2009 clinical evidence review on radiofrequency ablation of hepatic metastases from colorectal cancer. J Clin Oncol.

[6] Ricke J, Wust P, Wieners G, Beck A, Cho CH, Seidensticker M (2004). Liver malignancies: CT-guided interstitial brachytherapy in patients with unfavorable lesions for thermal ablation. J Vasc Interv Radiol.

[7] Ricke J, Wust P, Stohlmann A (2004). CT-guided brachytherapy. A novel percutaneous technique for interstitial ablation of liver metastases (in German). Strahlenther Onkol.

[8] Ricke J, Seidensticker M, Lüdemann L, Pech M, Wieners G, Hengst S, Mohnike K, Cho CH, Hänninen EL, Al-Abadi H, Wust P (2005). In vivo assessment of the tolerance dose of small liver volumes after single-fraction HDR irradiation. Int J Radiat Oncol Biol Phys.

[9] Ricke J, Mohnike K, Pech M, Seidensticker M, Rühl R, Wieners G, Gaffke G, Kropf S, Felix R, Wust P (2010). Local response and impact on survival after local ablation of liver metastases from colorectal carcinoma by computed tomography–guided high-dose-rate brachytherapy. Int J Radiat Oncol Biol Phys.

[10] Rühl R, Lüdemann L, Streitparth F, Seidensticker M, Mohnike K, Pech M, Wust P, Ricke J (2010). Radiobiological restrictions and tolerance doses of repeated single-fraction hdr-irradiation of intersecting small liver volumes for recurrent hepatic metastases. Radiat Oncol.

[11] Robin TP, Raben D, Schefter TE (2018). A contemporary update on the role of stereotactic body radiation therapy (SBRT) for liver metastases in the evolving landscape of oligometastatic disease management. Semin Radiat Oncol.

[12] Thames HD, Hendry JH (1987). Fractionation in radiotherapy.

[13] Lyman JT (1985). Complication probability as assessed from dose-volume histograms. Radiat Res.

[14] Burman C, Kutcher GJ, Emami B, Goitein M (1991). Fitting of normal tissue tolerance data to an analytic function. Int J Radiat Oncol Biol Phys.

[15] Kutcher GJ, Burman C, Brewster L, Goitein M, Mohan R (1991). Histogram reduction method for calculating complication probabilities for three-dimensional treatment planning evaluations. Int J Radiat Oncol Biol Phys.

[16] Marks LB, Yorke ED, Jackson A (2010). Use of normal tissue complication probability models in the clinic. Int J Radiat Oncol Biol Phys.

[17] Emami B, Lyman JT, Brown A (1991). Tolerance of normal tissue to therapeutic irradiation. Int J Radiat Oncol Biol Phys.

[18] Pan CC, Kavanagh BD, Dawson LA, Li XA (2010). Radiation-associated liver injury. Int J Radiat Oncol Biol Phys.

[19] Hall EJ, Giaccia AJ (2019). Radiobiology for the radiologist.

[20] Ricke J, Wust P (2011). Computed tomography–guided brachytherapy for liver cancer. Semin Radiat Oncol.

[21] Herfarth KK, Debus J, Lohr F, Bahner ML, Rhein B, Fritz P, Höss A, Schlegel W, Wannenmacher MF (2001). Stereotactic single-dose radiation therapy of liver tumors: results of a phase I/II trial. J Clin Oncol.

[22] Klement RJ (2017). Radiobiological parameters of liver and lung metastases derived from tumor control data of 3719 metastases. Radiother Oncol.

[23] Nicosia L, Cuccia F, Mazzola R, Figlia V, Giaj-Levra N, Ricchetti F, Rigo M, Bonù M, Corradini S, Tolia M, Alongi F (2020). Stereotactic body radiotherapy (SBRT) can delay polymetastatic conversion in patients affected by liver oligometastases. J Cancer Res Clin Oncol.

[24] Mazzola R, Fersino S, Alongi P, Di Paola G, Gregucci F, Aiello D, Tebano U, Pasetto S, Ruggieri R, Salgarello M, Alongi F (2018). Stereotactic body radiation therapy for liver oligometastases: predictive factors of local response by 18F-FDG-PET/CT. Br J Radiol.

